# Youth in the Netherlands Study (JOiN): study design

**DOI:** 10.1186/1471-2458-12-350

**Published:** 2012-05-14

**Authors:** Anja C Huizink, Kirstin Greaves-Lord, Brittany E Evans, Anja S Euser, Jan van der Ende, Frank C Verhulst, Ingmar HA Franken

**Affiliations:** 1 VU University, Faculty of Psychology and Education, Department of Developmental Psychology, van der Boechorststraat 1, 1081 BT, Amsterdam, the Netherlands; 2 Department of Child and Adolescent Psychiatry, Erasmus Medical Center, P.O. Box 2060, 3000 CB, Rotterdam, the Netherlands; 3 Institute of Psychology, Erasmus University Rotterdam, Woudestein T12-59, P.O. Box 1738, 3000 DR, Rotterdam, the Netherlands

**Keywords:** Adolescent Substance Use, Endophenotypes, EEG, Stress reactivity, Behavioral Disinhibition

## Abstract

**Background:**

Adolescence is a critical developmental period regarding exposure to substances. Therefore, it is important to be able to identify those adolescents who are most vulnerable to substance abuse in the (near) future. The JOiN study was specifically designed to examine two endophenotypes of adolescent substance use in a normal risk (NR) and high risk (HR) sample of adolescents: (1) behavioural disinhibition, and (2) individual differences in stress sensitivity.

**Methods:**

The NR adolescents were part of a longitudinal general population study at the Erasmus Medical Center in Rotterdam, the Netherlands of children and adolescents initially aged 6 to 18 years old. Three assessment waves have been nearly completed, and data are available of N = 711 participants for stress sensitivity measures, and of a subsample of N = 110 for electroencephalography (EEG) measures. Added to this study, HR adolescents who had at least one parent with a substance use disorder and who were treated by an outpatient clinic of a primary addiction care provider were approached via their parent(s). In total, N = 83 adolescents formed this HR sample. NR and HR adolescents participated in standardized stress procedure and EEG procedures in our laboratory. Questionnaires were filled out on background variables, behavioural and emotional problems, and substance use, and a diagnostic interview was conducted with adolescents and parents to assess psychopathology symptoms. DNA was collected through saliva or blood samples.

**Discussion:**

The design of the JOiN study is optimal for examining the predictive role of endophenotypes of adolescent substance use. The combination of different methods, i.e. stress physiology, electrophysiology, genetics, and questionnaire data from several informants on a range of behaviours and environmental factors enables the investigation of the multifactorial nature of adolescent substance use.

## Background

Since 1983, the Department of Child and Adolescent Psychiatry in Rotterdam, the Netherlands, has conducted surveys every decade to assess the level of psychiatric problems in children and youth, to test for time-trends in the occurrence of these problems, and to (re)validate the well-known and often used ASEBA instruments in the Netherlands (South Holland study). Numerous publications can be found that describe the findings of these surveys (e.g. [1-5]). As part of that ongoing study, the Youth in the Netherlands (*Jongeren Onderzoek in Nederland* in Dutch, abbreviated as JOiN) study was initiated. This study selected adolescents from the survey, aged 12 to 20 years, and enriched this sample with high risk (HR) youth, of whom at least one parent was in clinical treatment for a substance use disorder (alcohol or illicit drugs).

### Scope of research

Adolescence is a critical developmental period regarding exposure to substances. Therefore, it is important to be able to identify those adolescents who are most vulnerable to substance abuse in the (near) future. Genetic susceptibility is one important factor that underlies individual vulnerability. For complex multifactorial disorders such as substance abuse, the identification of susceptibility genes may be substantially aided by using quantitative intermediate phenotypes, or endophenotypes, instead of dichotomized clinical variables [6]. These endophenotypes reflect neurobiological pathways to substance abuse. The JOiN study was specifically designed to examine several endophenotypes of adolescent substance use. These endophenotypes represent two neurobiological pathways to substance misuse or abuse: (1) behavioural disinhibition, as a possible result of altered cognitive functions of the prefrontal cortex and (2) individual differences in stress sensitivity. Behavioural disinhibition includes two measures: (a) the P300 component of the Event-Related Potential (ERP), which is associated with attentional allocation and context updating processes of working memory and involves the activation of inhibitory processes [7], and (b) risky decision making, or reward-associated decision-making skills, which have been associated with substance abuse in adults. Likewise, individual differences in stress physiological responses are represented by two endophenotypes: measures of stress reactivity of (c) the hypothalamic-pituitary-adrenal (HPA) axis and (d) the autonomic nervous system (ANS). More details on the measures of the endophenotypes and the outcomes are presented in Tables 1 and 2, respectively.

**Table 1:** Endophenotypes under study

**Behavioural disinhibition**		
*Endophenotype*	*Abbreviation(s)*	*Assessment*
(In)efficient allocation of attentional resources in processing task-relevant cognitive information, indexed by the P300 amplitude of the Event-Related brain Potential of an Electroencephalography	P300, ERP, EEG	Visual novelty oddball paradigm to elicit P300 ERP response [8-11].
Reward-associated/Risky decision-making skills	FRN, P300	Balloon Analogue Risk Task (BART; [11,12]) Personality measures by means of BIS/BAS scales [13], BSSS [14], and I^7^[15,16].
**Stress sensitivity**		
*Endophenotype*	*Abbreviation(s)*	*Assessment*
Stress reactivity of the hypothalamic-pituitary-adrenal axis, indexed by cortisol	HPA	Cortisol responses to laboratory stressors, including public speaking, high speed mathematics, mental arithmetic tasks [17,18].
Stress reactivity of the autonomic nervous system, including blood pressure, heart rate, heart rate variability responses	ANS, BP, ECG, HR, HRV	ANS responses to laboratory stressors, including public speaking, high speed mathematics, mental arithmetic tasks [17,18].

**Table 2:** Main outcomes of the JOiN study assessed at T1 and/or T2

**Behaviour**	**Data-wave**	**Instrument**
Alcohol use disorder: symptoms of abuse and dependence	T1	Adolescent: DISC [19], Parent: CIDI [20].
Cannabis use disorder: symptoms of abuse and dependence	T1	Adolescent: DISC [8,19], Parent: CIDI [20].
Other substance use disorder: symptoms of abuse and dependence	T1	Adolescent: DISC [8,19], Parent: CIDI [20].
Ever use of nicotine, alcohol, cannabis or other drugs	T1, T2	Self-reported Substance Use Questionnaire
Age of onset of nicotine, alcohol, cannabis or other drugs	T1, T2	Self-reported Substance Use Questionnaire
Frequency and quantity of use of nicotine, alcohol, cannabis or other drug use	T1, T2	Self-reported Substance Use Questionnaire
Level of drunkenness or being stoned last time alcohol or cannabis were used	T1, T2	Self-reported Substance Use Questionnaire

The general aims of the JOiN study are:

(1) to examine the role of endophenotypes, representing two potentially important underlying pathways towards substance abuse, that explain individual resilience and vulnerability to early onset of substance use and the transition to regular use;

(2) to identify, based on the endophenotypes, the most vulnerable group of adolescents with regard to substance misuse and abuse;

(3) to examine the relationship between endophenotypic characteristics and common and specific DNA polymorphisms that have been associated with increased risk for substance abuse in adult populations.

## Methods/design

The JOiN study sample consists of a *normal risk* (NR) sample and a *high risk* (HR) sample. The NR sample is part of a larger sample that participated in a longitudinal general population study at the Erasmus MC in Rotterdam, the Netherlands of children and adolescents initially aged 6 to 18 years old [21]. For this larger study, 2567 children and adolescents were randomly drawn from municipal registers of 35 representative municipalities in the Dutch province of South Holland including urban and rural areas. Of these 2567 children and adolescents, 2286 were eligible for participation (191 had parents that did not speak Dutch, 31 had a physical or mental disability, 31 were unable to be reached (had missing or faulty contact details), 22 departed the study area, 6 for whom no person could complete questionnaires). Of the 2286 eligible participants, 1710 (74.8%) agreed to participate at the baseline assessment wave (T0), which took place in 2003–2004. Participants did not differ from non-participants at this baseline assessment in terms of gender (*p* > .05). The next assessment wave (T1) of this sample ran from September 2005 until March 2009. A second follow-up (T2) started in 2009 and is currently nearly completed.

**Figure 1:**
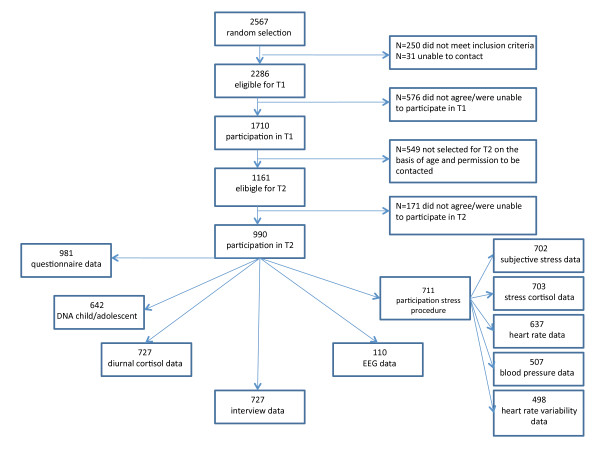
**Flow chart of participation of the*normal risk*(NR) population**

The JOiN study includes a subgroup of participants of this larger study that took part in a stress and electroencephalography (EEG) procedure at T1 (see Figure 1), which we refer to as the *normal risk* (NR) sample. Of the 1710 participants at T0, n = 1161(67.9%) participants were eligible to be part of this NR population, the other n = 549 were not selected based on their age (< 11 years old) or lack of permission to be contacted for follow-up research. Of these 1161 eligible individuals, N = 990 (85.3%) participated in T1, of which N = 711 in the stress procedure, and N = 110 in the EEG procedure. For more details on the NR population, please see the section ‘Response’ further down below and Figure 1.

We further enriched this NR sample with a *high risk* (HR) population of adolescents. The HR population of adolescents included participants with a familial risk for substance use disorders. These HR adolescents presumably have an increased risk to show altered endophenotypic characteristics due to their genetic vulnerability for substance use disorders. They all had at least one parent with a substance use disorder (alcohol or illicit drugs), for whom treatment was necessary and provided by Bouman GGZ, the primary addiction care provider in the city of Rotterdam and the surrounding area (Zuid-Holland), and were considered as HR for our study purpose. The parent(s) of these adolescents were recruited from a consecutive series of patients referred to Bouman GGZ in the period of May 2009 – November 2011. For a small number of HR participants (n = 6) of whom the parent had a substance use disorder, the parent was not in treatment, but parent and adolescent were recruited by word of mouth. For these parents, substance use disorder was ascertained with a standardized diagnostic interview (CIDI; [20]) prior to participation of the adolescent in the study. In total, N = 83 HR adolescents were included in the JOiN study. Other inclusion criteria for this HR group of adolescents were similar to those of the NR population. We specifically checked for (severe) mental retardation and epilepsy, which were considered exclusion criteria for EEG measures.

### Power of the study

Our sample size was calculated to be at least N = 600 in order to have enough power to run our analyses. For continuously distributed variables (such as number of cigarettes smoked, number of alcoholic beverages consumed), such a sample size yields a power of 0.99 to show a contribution to the explained variance of 5% in a linear regression model with 10 predictors, for main effects and/or interactions (alpha set on 0.05). For categorical outcome variables with a rate of 12%, a sample size of N = 600 yields a power of > 90% to demonstrate a Relative Risk (RR) of 2 (alpha set on 0.05). Because the study has several measurements on behavior and substance use repeated over time, the accuracy of measuring the true underlying value will be increased, which will add to the statistical power for these measurements. Also, the addition of a high risk sample of adolescents will increase the frequency of risk alleles in our total study sample. The program Quanto V was used for genetic power analyses for continuous outcomes (e.g. number of cigarettes smoked). For the estimate of power to detect main effects of genes, we assumed a ‘disorder’ allele frequency of 0.35, which is based on actual allele frequencies of DRD2 and DRD4 SNPs that were genotyped in a Finnish Twin study, very recently (Kaprio, personal communication). For continuous outcomes, the study was estimated to have over 80% power to detect a genetic main effect and a G*E interaction effect that accounts for 10% or more of the total variance.

### Study procedure and data collection

The study was approved by the Medical Ethics Committee of the Erasmus Medical Center, the Netherlands, and was conducted in accordance with the declaration of Helsinki. Written informed consent was obtained from all participating adolescents and their parents.

Participants for the NR population were informed about the study through mail sent to their home address, and a subsequent telephone call to invite them to participate in the baseline assessment (T0, [21]). Data for T0 were collected via mailed packages containing questionnaires sent to the participants and their parents. If the participants agreed to participate, but did not return their questionnaires in time, they were reminded through a telephone call from the researchers for several times (maximally 3) if needed. Data from T0 were scanned using Teleform into data-files and were visually checked for missing data or errors by the researchers.

NR adolescent participants at T0 were approached by telephone by our researchers for the T1 assessment and the general goals of the JOiN study were explained. Appointments were made for a visit to (a) the Erasmus Medical Center or a testing facility near to the participants’ home address and (b) the Erasmus Behavioral Lab (Erasmus University Rotterdam) to measure the endophenotypes under study in two experimental sessions. The NR adolescents first participated in a stress procedure, conducted at a laboratory of the Erasmus Medical Center, Department of Child and Adolescent Psychiatry. One hundred and ten of these NR adolescents (native Dutch adolescents, 59 males, ranging from 12 to 20 years old, mean age = 15.26, SD = 2.17) subsequently participated in an EEG procedure at the Erasmus Behavioral Lab (Erasmus University Rotterdam). More details about these EEG procedures are provided below.

The parents of the HR population were informed about the study by the treatment staff of Bouman GGZ. All patients who had a child in the age range of 12–20 years received an information package on the study, and were asked if they approved of their child to participate. If they agreed, the patients and their child were screened by telephone on inclusion and exclusion criteria. If they were found to be eligible, an appointment was made for T1 assessments. 107 families were approached which included at least 131 eligible adolescents. Of these, 83 adolescents from 64 families participated in the study.

Prior to the experimental sessions (see below), questionnaires and four tubes for assessment of salivary cortisol levels on a normal day were sent to the participants by mail. Detailed written instructions were given on the time and manner of sample collections, and to preserve the tubes in the freezer until the testing day. Participants were instructed to provide the first sample directly upon awakening (Cort1), the second 30 minutes afterwards (Cort2), the third at 12 p.m. (Cort3) and the fourth at 8 p.m. (Cort4). Participants then brought the frozen tubes to the session that collected their endophenotypic characteristics, along with questionnaire data on the main outcomes and covariates. The experimental sessions included a stress procedure and an EEG procedure, which together lasted approximately three to four hours.

The *stress procedure* took place during the afternoon in order to minimize differences in cortisol levels due to normal diurnal variations. The experiment leader explained the procedure before the session started. The electrodes of the electrocardiogram were attached and participants were told to breathe normally and to relax. For the NR population, this procedure started with filling out a questionnaire set, followed by a ten minute pre-task resting period, after which the social stress tasks began. For the HR sample, the questionnaire set was filled out shortly before the EEG procedure, but the stress procedure similarly started with a ten minute pre-task resting period. The stress session ended with a five minute recovery period and a subsequent calm nature documentary that was shown for 25 minutes for further relaxation. After each period/task, during the middle of the movie and at the end of it, the participant was asked to passively drool into a test tube in order to collect salivary cortisol. In total, six test tubes were filled with saliva on the day of the testing session, and heart rate was monitored continuously using a three-lead electrocardiogram throughout the entire stress procedure, which lasted for approximately 90 minutes. Also, blood pressure was monitored continuously, using a Portapress device. All saliva samples were kept in a freezer at −20 degrees Celsius until data collection was complete. All samples were sent to the laboratory (Kirschbaum Laboratory in Dresden, Germany) for analysis. A time-resolved fluorescence immunoassay was used in order to determine the cortisol concentration in the saliva samples. Heart rate data were stored offline to be analyzed at a subsequent moment and were visually checked for artefacts.

The *EEG session* lasted approximately 75 minutes in total. Participants were seated on a comfortable chair in a light and sound-attenuated room. While filling in a questionnaire set, the EEG electrodes were attached. Hereafter, participants conducted a visual novelty oddball task and a gambling/decision-making paradigm (i.e., the Balloon Analogue Risk Task; BART).

The EEG was recorded with BioSemi Active-Two using 34 scalp sites (10–10 system, and two additional electrodes at FCz and CPz) with Ag/AgCl active electrodes mounted in an elastic cap. Two electrodes were attached to the left and right mastoids as reference electrodes. To be able to correct for ocular artifacts, two electrodes were placed next to each eye for horizontal electrooculogram (HEOG) and two electrodes were placed above and below the left eye for vertical electrooculogram (VEOG). Online signals were recorded with a low-pass filter of 134 Hz. All signals were digitized with a sampling rate of 512 Hz and 24 bit A/D conversion. EEG data were stored offline to be analyzed (using Brain Vision Analyzer 2) at a subsequent moment and then off-line referenced to mathematically linked mastoids, filtered, ocular corrected, baseline corrected, visually checked for artefacts and finally, relevant epochs were averaged for relevant artefact free trials at each scalp site, for each participant. All participants received a gift certificate worth 50 euro for their participation in the experimental session.

Besides questionnaire data and endophenotypic data, *DNA* was also collected at T1 through saliva or blood samples from the adolescents. Blood and buccal swab samples were stored at −20 degrees Celsius. Saliva samples contained in Oragene containers were stored at room temperature. Genotyping will be conducted by PCR (polymerase chain reaction) in a genetic laboratory.

Furthermore, *diagnostic interview data* were collected at T1. Parents of the participants who had completed the experimental sessions were contacted by telephone by a research assistant to make an appointment for interviews with the parents as well as the adolescents. The Composite International Diagnostic Interview (CIDI; [20]) pertains to symptoms of psychopathology and was completed by either one or both parents 73.4% (727/990) participated in the interviews, of which 35.2% mother, 5.0% father, 59.8% both). The Diagnostic Interview Schedule for Children (DISC; [19,22]) pertains to symptoms of psychopathology in youth and was completed by one of the parents about their child (parent-report version) and by the participants about themselves (self-report version).

Finally, T2 assessment for the NR and HR adolescents was conducted approximately one year after T1 through mailed packages of questionnaires (see procedure for T0).

### Data collection normal risk population only

At baseline (T0, 2003–2004) general demographic information (i.e. gender, age, social-economic status, and ethnicity) was collected from the sample of NR participants of the general population as well as information on behavioural and emotional problems. To obtain a complete view on adolescent’s problem behaviours, information from multiple informants and differences among them is needed [23]. Adolescents’ behavioural and emotional problems were assessed with: Child Behaviour Checklist (CBCL/6-18; parent report); Youth Self-Report (YSR); Teachers’ Report Form (TRF). The content of the above mentioned scale is the same for all three informants, although the scales vary in some items across informants. All items, which are scored 0 (not true), 1 (somewhat or sometimes true) or 2 (very true or often true), are based on the preceding 6 months. Eight syndrome scores are derived: Withdrawn/Depressed, Somatic Complaints, Anxious/Depressed, Social Problems, Thought Problems, Attention Problems, Rule-Breaking Behavior, and Aggressive Behaviour. Good reliability and validity of the instruments have been replicated for the Dutch translation [24-26].

### Data collection at T1: normal risk and high risk population

#### Predictor variables

##### Genotype

Polymorphisms in several genes related to our endophenotypes and/or our outcome of interest will be determined, including (but not restricted to) the D2/4 (DRD2/DRD4) dopamine receptor gene, the mu-opioid receptor gene (OPRM1), the GABA-a receptor gene (GABRA2), the muscarinic acetylcholine receptor gene (CHRM2), and the Catechol-O-Methyltransferase (COMT) gene.

##### Endophenotypes

An overview of endophenotypes covered in the JOiN study is provided in Table 1. For the first neurobiological pathway, *behavioural disinhibition*, several cognitive indices and personality measures were included in our study design. We used a visual novelty oddball paradigm to elicit a P300 ERP response. The amplitude of the P300 has been proposed to reflect attentional allocation, with reduced P300 amplitudes referring to inefficient allocation of attentional resources in processing task-relevant cognitive information (e.g. [8,9]). Furthermore, the automatic response mode version of the Balloon Analogue Risk Task (BART; see [12,27] for further details) was used as an index of reward-associated/risky decision-making skills. In addition, by pairing this measure with EEG recordings, we were also able to investigate the brain’s feedback processing mechanisms during risky decision-making (i.e., the feedback-related negativity (FRN) and the feedback-related P300 amplitude). Moreover, personality traits encompassing three related personality traits of impulsivity, sensation seeking tendencies and sensitivity for reward were assessed, using the I^7^ questionnaire [15,16], the Brief Sensation Seeking Scale (BSSS; [14]) and BIS/BAS scales [13,28], respectively. The second neurobiological pathway, *individual sensitivity to stress*, is measured in a standard laboratory setting. Three tasks are used to elicit a physiological stress response of the participants, after baseline measures are collected. Physiological stress responses include cardiovascular reactivity measures (blood pressure, electrocardiogram), and hypothalamic-pituitary-adrenal (HPA) axis response (cortisol). Tasks include (1) a public speaking task, (2) a high speed mathematics task on the computer, and (3) a mental arithmetic task. These tasks have repeatedly been shown to elicit stress responses (e.g. [17,18,29]).

##### Covariates

General background characteristics were collected on gender, SES, age, weight and height (to determine Body Mass Index), pubertal stage by Tanner [30,31], oral contraceptive use (females only), medication use, birth outcome information reported by the parent(s).

Extensive measures of symptoms of psychopathology were collected in both NR and HR adolescents, including: CBCL, YSR, TRF (see T0), Antisocial Process Screening Device (APSD; [32]); Multidimensional Anxiety Scale for Children (MASC; [33]). In addition, information on temperament was collected with the Emotionality Activity Sociability scale (EAS; [34]). Occurrence of life events were also assessed using a self-report questionnaire developed by the TRAILS consortium [35,36].

Information on family and parental factors included parental substance use (reported by the parent(s)), including information on parental substance use during pregnancy, parenting style as reported by the adolescent and parent (‘My memories of upbringing-Child; EMBU’ [37]) and the ‘Alabama Parenting Questionnaire’; [38]).

##### Outcome variables

An overview of the most important outcomes for the study aims of the JOiN study is listed in Table 2. To determine symptoms of alcohol, cannabis and other drug abuse and dependence, based on DSM- IV criteria, a highly structured respondent-based interview, the NIMH Diagnostic Interview Schedule for Children (DISC) was applied. Unless otherwise specified, the timeframe of the DISC is the past 12 months. The DISC has two parallel forms: DISC-C administered directly to the adolescent, and DISC-P administered to the parent or parent substitute. Both are used in the present study. The reliability and validity of the DISC have been supported by previous studies [19,22]. Trained and certified students administered the DISC.

To assess age of onset of substance use of adolescents, frequency of substance use, quantity of use, and level of drunkenness or being stoned, a self-reported Substance Use Questionnaire was used, including items on (1) age at first use, (2) (a) monthly, yearly and lifetime prevalence of use, and (b) monthly, yearly, and lifetime prevalence of intensive use (i.e. drunkenness for alcohol users); (3) (a) quantity of alcohol use during the week, and (b) during weekends; (4) (a) number of weekdays and (b) number of weekend days in which alcohol was consumed; (5) the level of drunkenness, and being stoned the last time alcohol or cannabis was consumed, respectively.

### Response

#### Normal risk population

The response rates for the NR sample are illustrated in Figure 1 (flowchart). The 990 individuals that took part in T1 did not differ from those who did not (n = 171) with regard to gender or psychopathology, although they did have a slightly higher social economic status (*p* < .001). The 711 individuals that participated in the stress procedure did not differ from those who did not (n = 990 – 711 = 279) regarding T0 psychopathology, but they did show somewhat more internalizing problems during T1 (*p* < .05) and more girls participated than boys (*p* < .01). No differences were found between the 110 individuals that participated in the EEG experiment and those who did not.

#### High risk population

Estimation of the precise number of eligible adolescents for the HR population, of whom at least one parent was a patient at Bouman GGZ from May 2009 until November 2011 is difficult, because there was no satisfactory registry of the number or age of children of this patient group of Bouman GGZ at the start of our study. Therefore, we did not attempt to identify the overall response rate of this population. However, several remarks regarding the response of this particular subsample can be made. First, when patients at Bouman GGZ were approached by our researchers or by their counsellors to ask for their approval to have their adolescent child contacted by our researchers, we noticed that a large number of patients had limited contact or disturbed relations with their child. This first hurdle reduced the number of available adolescents for our study. Second, if the parent agreed to have their child contacted by our researchers, some of the adolescents themselves did not want to be confronted with the addiction problem of one of their parents, and refused to participate for that reason, or did not have time or motivation to take part in the extensive measures. Third, perhaps because of the adolescent age of our population, if arrangements were made to participate in our study, about half of the adolescents were no-shows at the set time and date for their assessment. Nonetheless, of the 107 approached families, we were able to include n = 83 participants in the HR population.

## Discussion

### Limitations

As described in the response section, there was a slight response bias in the NR population with more female, higher socioeconomic status individuals participating. Since children whose parents did not speak Dutch were excluded, the group of ethnic minorities is underrepresented. Moreover, to limit the amount of time spent by the adolescents and their parent(s) in order to maintain feasibility and keep the burden for participants acceptable, we did not include all measures that might, in retrospect, be useful for examining additional hypotheses in our study. For instance, although we included information on several personality characteristics, and on various symptoms of psychopathology, we did not include the whole spectrum of personality that might underlie the occurrence of risk taking behaviour such as substance use. Thus, the breadth of focus may be limited, but the counterbalance is offered by detailed information collected on several occasions on other important factors. Another weakness of the study is that some possibly important determinants of psychopathology that operate earlier in life, such as birth outcomes and prenatal influences, have been assessed retrospectively or not at all.

### Strengths

The longitudinal design of the JOiN study, in which a normal risk population of adolescents is combined with a high risk population of adolescents with a familial vulnerability for substance use disorders, is optimal for examining the predictive role of endophenotypes of adolescent substance use.

Furthermore, the combination of different methods, i.e. stress physiology, electrophysiology, genetics, and questionnaire data from several informants on a range of behaviours and environmental factors, in the JOIN study, enables the investigation of the multifactorial nature of adolescent substance use. In addition, the unique element of our study is that various interrelations among endophenotypic characteristics, and between genotype and endophenotype can be tested for their predictive value of substance use. It may therefore contribute to the identification of adolescents at risk for onset of substance use and those who progress to regular use and abuse.

## Abbreviations

JOiN: Youth in the Netherlands Study [in Dutch]; NR: Normal risk; HR: High risk; EEG: Electroencephalography; ERP: Event-related potential; HPA axis: Hypothalamic-pituitary-adrenal axis; ANS: Autonomic nervous system; BART: Balloon analogue risk task; T0: Baseline assessment of normal risk population; T1: Assessment of endophenotypes in both normal risk and high risk population; T2: Follow-up assessment one year after T1.

## Competing interest

Dr. Verhulst is a contributing author of the Achenbach System of Empirically Based Assessment, from which he receives remuneration. No other competing interests are reported.

## Authors’ contributions

ACH conceived of the study, participated in its design and supervised the researchers involved of acquisition of data, acquired the funding, and drafted the manuscript. KGL participated in the coordination of the data-collection and participated in drafting the manuscript. BE and AE substantially contributed to acquisition of data and analysis and interpretation of descriptive data. JvdE and FCV were involved in the conception of the study, participated in its design and critically reviewed drafts of the manuscript. IHAF participated in the conception of the study, contributed to supervision of the researchers involved and critically reviewed drafts of the manuscript. All authors read and approved the final manuscript.

## Pre-publication history

The pre-publication history for this paper can be accessed here:

http://www.biomedcentral.com/1471-2458/12/350/prepub
